# Methamphetamine abuse impairs sequential working memory

**DOI:** 10.3389/fpsyt.2024.1458509

**Published:** 2024-09-27

**Authors:** Wang Yao, Hao Zhang, Shuaiqi Li, Sensen Song, Zheng Ye, Xiaolin Zhou

**Affiliations:** ^1^ School of Psychological and Cognitive Sciences and Beijing Key Laboratory of Behavior and Mental Health, Peking University, Beijing, China; ^2^ School of Education and Psychology, Chengdu Normal University, Chengdu, China; ^3^ School of Psychology, Central China Normal University, Wuhan, China; ^4^ Institute of Neuroscience, Center for Excellence in Brain Science and Intelligence Technology, Chinese Academy of Sciences, Shanghai, China; ^5^ School of Psychology and Cognitive Science, Shanghai Key Laboratory of Mental Health and Psychological Crisis Intervention, East China Normal University, Shanghai, China

**Keywords:** working memory, sequential working memory, cognitive deficits, methamphetamine dependence, digit ordering task

## Abstract

The ability to maintain and manipulate sequential information in working memory, referred to as sequential working memory, plays a vital role in our daily life. While research has shown that methamphetamine abuse affects the neural substrates and the overall functioning of working memory, its specific impact on sequential working memory remains unclear. In this study, we asked 62 abstinent methamphetamine-dependent participants and 59 control participants to complete a digit ordering task in which they saw four digits one-by-one over time and subsequently rearranged them in ascending order. The four digits were presented either randomly in the experimental condition or in ascending order in the control condition. Results show that methamphetamine-dependent participants performed worse than the controls in the experimental condition in which sequential working memory was needed to complete the task, but not in the control condition in which only short-term memory was needed. This finding demonstrates that methamphetamine abuse impairs sequential working memory.

## 1 Introduction

Methamphetamine is a potent stimulant that exerts significant effects on the central nervous system. It has been found that the abuse of methamphetamine can lead to significant and enduring neurotoxicity and neurodegeneration in individuals (1), including the disruption of dopaminergic, serotonergic, and noradrenaline neurotransmission systems (2, 3). The disruption of neurotransmission is linked to functional and structural irregularities in various brain regions, such as the prefrontal cortex, striatum, mesolimbic system, and hippocampus (4, 5). Given that these regions play a crucial role in a number of cognitive functions, including working memory (6, 7), it is reasonable to assume that methamphetamine abuse could have profound impacts on cognition.

Indeed, extensive evidence documents impairments of working memory by methamphetamine abuse, in the n-back task, digit span task, delayed match to sample task, spatial working memory task, among others (7–11). However, it is unclear to what extent the functioning of sequential working memory is also affected by methamphetamine abuse.

Sequential working memory refers to the ability to maintain and manipulate the order of items in working memory, a function that differs from the mere storage of information (i.e., short-term memory) related to individual items, such as their identity, pattern, or color. This ability is essential for various cognitive processes, encompassing verbal activities such as speech perception and spelling, as well as nonverbal activities like planning and goal-directed behavior (12, 13). The neural substrates underlying sequential working memory have been identified to involve the lateral prefrontal cortex, basal ganglia, and intraparietal sulcus (13–17). These regions’ functions are susceptible to impairment due to methamphetamine abuse (4, 18). Consequently, it is plausible that methamphetamine abuse could impact sequential working memory in affected individuals.

Two earlier studies employing a sequential working memory task, however, failed to yield findings supporting this hypothesis (19, 20). In these studies, the researchers utilized a Letter-Number Sequencing task adopted from the Wechsler Adult Intelligence Scale (21), where participants were exposed to a randomly arranged sequence comprising a combination of letters and numbers. No significant difference in performance was observed between the methamphetamine-dependent participants and the normal controls when they were tasked with separately reporting the letter and number sequences in either ascending or descending order. Due to the limited number of participants included in the two studies (N of methamphetamine-dependent participants = 16 and 22, respectively), it is necessary to re-evaluate the null effect. Moreover, the Letter-Number Sequencing task does not possess the capability to distinguish sequential working memory from other cognitive functions, such as working memory span.

In the current study, a substantial number of participants were examined using an enhanced sequential working memory task. During this task, participants were presented with four unique numerical digits in sequence and subsequently required to recall the digits in ascending order following a delay. Crucially, the digits presented were either in a random order or in ascending order. The former condition requires participants to re-order the digits in working memory, while the latter does not (13). Incorporating an ordered (control) condition into the experimental design would enable us to eliminate confounding variables in cognitive functions beyond sequential working memory, such as working memory span.

## 2 Materials and methods

### 2.1 Participants

We enrolled 62 abstinent individuals dependent on methamphetamine (32 females, M_age=27.3 ± 4.2 years, M_education=9.4 ± 1.5 years) who met the diagnostic criteria for substance use disorder according to the DSM-5 (27) from the Addiction Rehabilitation Center of Sichuan province, China. Additionally, we posted recruitment advertisements in the local community and included 59 healthy controls (29 females, M_age = 35.6 ± 5.8 years, M_education = 10.2 ± 1.5 years) who responded from the local community. Participant demographics are summarized in **Table 1**. At the time of testing, the participants dependent on methamphetamine had not utilized any medication for addiction rehabilitation. The study was reviewed and approved by the Ethics Committee of the Sichuan Psychological Society, and informed consent was obtained from the participants.

**Table 1:** Participants’ demographic information and methamphetamine use characteristics.

Features	Methamphetamine Group	Control Group
Male/Female	30/32	30/29
Age (years)	27.3 (4.2)	35.6 (5.8)
Education (years)	9.4 (1.5)	10.2 (1.5)
Period of Drug Abuse (years)	5.29 (3.1)	Null
Period of abstinence (months)	12.9 (6.2)	Null

It is important to acknowledge that the control group comprised individuals who were older (95% CI of age difference = [6.47, 10.14], p < 0.001) and had received a higher level of education (95% CI of education difference = [0.24, 1.33], p < 0.01) than the methamphetamine group. In China, individuals with addictive behaviors typically have lower age and educational levels. However, due to the development of the education sector in China, there are not many people in the local community who match the methamphetamine group in both age and educational level. If we insist on using an age-matched control group, it will increase the educational level disparity between the two groups, while using an education-matched control group will increase the age disparity between the two groups. Ultimately, we relaxed the age restrictions during participant recruitment to ensure that the differences in age and educational level between the two groups were not too large. In the subsequent statistical analyses, we controlled for the differences in age and educational level between the two groups.

### 2.2 Experiment procedure

The experimental procedure was programmed using E-Prime 3.0 and conducted on a 17-inch laptop computer (screen resolution: 2240x1400, 60.02 Hz). Participants were brought into a standardized behavioral laboratory in batches, ensuring no communication or interaction among them to prevent any mutual interference. Each participant sat facing the computer, with a distance of 60 cm between their head and the computer screen.

**Figure 1:**
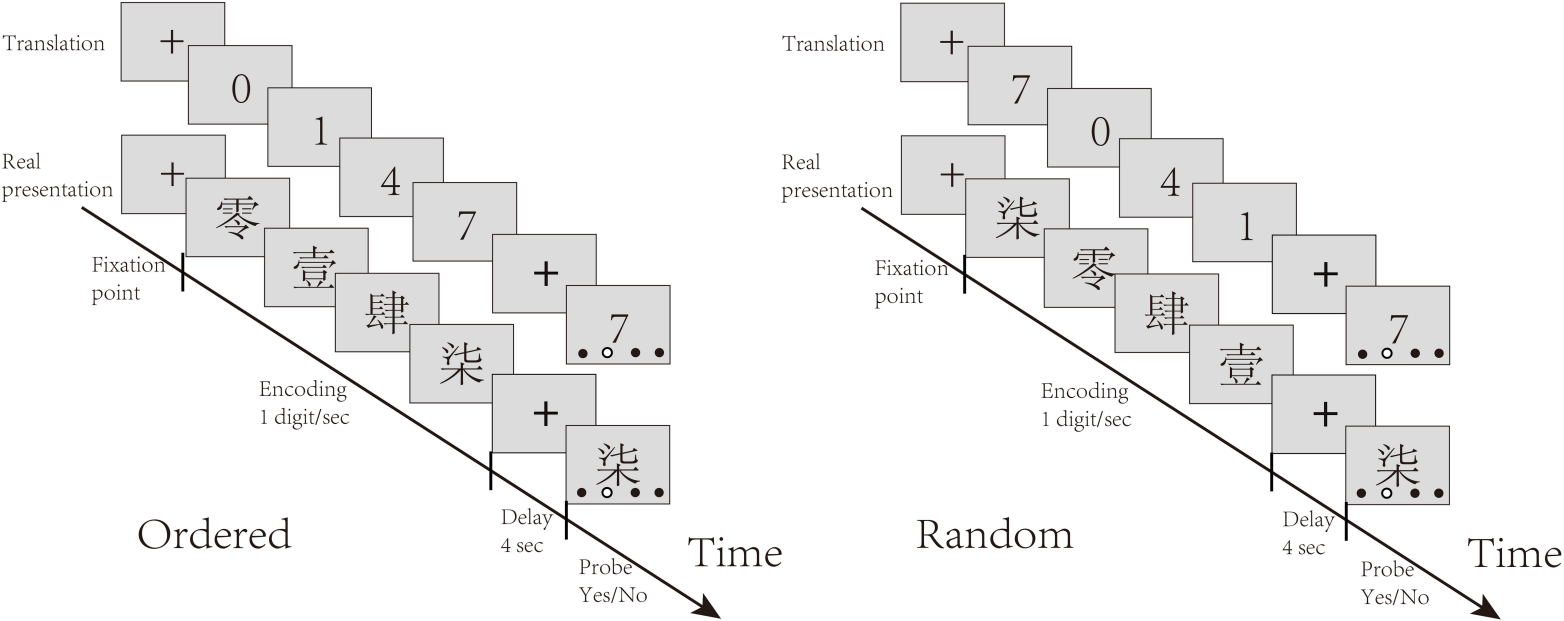
Task structure: The task included interleaved random and ordered conditions. In each trial, participants were presented with four distinct Chinese characters representing numbers one by one. They were required to remember the order of these numbers based on their magnitudes. In the ordered condition, the numbers appeared in ascending order, while in the random condition, the numbers were presented in a random order. After the delay, participants had to judge whether the hollow dot indicated the target position of the digit probe by pressing buttons with the right hand.

The experiment contained two blocks of stimuli, each containing 31 trials (15 ordered trials, 16 random trials). In each trial, participants were presented with four distinct digits, written in Chinese characters, sequentially at one digit per second (13). There were two conditions randomly interleaved in the task: the control (Ordered) condition, in which the digits were presented ascendingly, and the experimental (Random) condition, in which the digits were presented randomly. Participants were asked to remember the digits in ascending order through a short delay of 4 seconds. The screen then displayed one of the digits, along with four dots indicating four ordinal positions. The participants were asked to judge whether the digit matched the order of output indicated by the red dot. They had up to 5 seconds to respond by pressing keys. **Figure 1** illustrates the task procedure.

In the Ordered condition, the numerical order of the digits was inherently clear and only short-term memory was needed to store the digits. In the Random condition, participants needed to rearrange the digits in memory before reporting them in numerical order. The comparison between the Random and Ordered conditions served to isolate sequential working memory manipulation.

### 2.3 Data analysis

Trials with a reaction time (RT) shorter than 0.2 seconds or longer than 5 seconds were excluded from data analysis. A total of 29 trials (15 in the Methamphetamine Group), representing approximately 0.38% of the initial 7502 trials, were excluded from the analysis.

The RTs and response accuracy were analyzed using a linear mixed effects model. For the fixed effects, we included condition, group, and their interaction. For the random effects, we included a random intercept and a random slope for condition, for each participant. To control for the effects of age and level of education, we included them as covariates in the models. All data analyses were conducted using the stats models package (22) in Python.

## 3 Results

**Figure 2:**
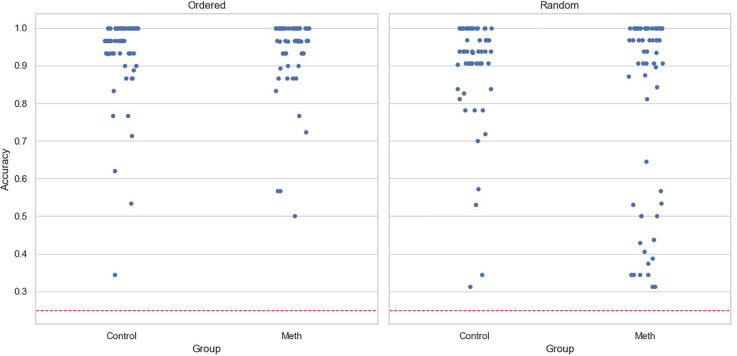
Task accuracy for each participant across conditions. The dashed line represents chance level, and each point represents one participant.

For accuracy data, the main effects of participant group (Coef=0.042, SE=0.03, p=0.16) and experimental condition (Coef=-0.031, SE=0.024, p=0.192) were insignificant. The interaction was significant (Coef=-0.11, SE=0.033, p=0.001). A simple effects test revealed that methamphetamine-dependent participants performed worse than the control group in the Random condition (F(1,119)=6.57,p< 0.05), but not in the Ordered condition (F(1,119)=0.35,p= 0.55). Notably, neither age (Coef=0.003, SE=0.002, p=0.201) nor level of education (Coef=0.009, SE=0.008, p=0.268) had significant effects. **Figure 2** shows the accuracy for participants under two conditions, with each point representing a participant.

**Figure 3:**
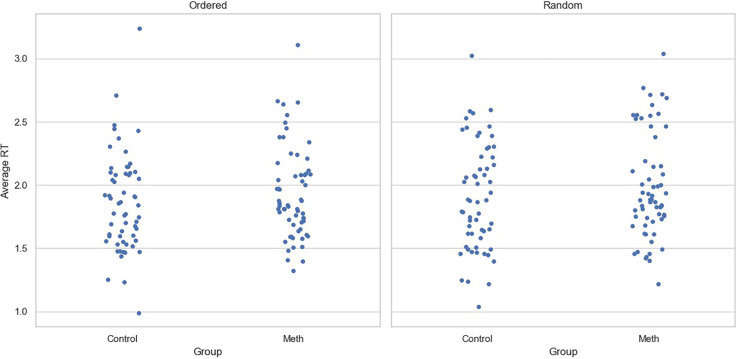
Average reaction time for each participant across conditions. Each point represents one participant.

For RTs, although the main effect of experimental condition was significant (Coef=0.049, SE=0.024, p=0.04), neither the main effect of participant group (Coef=0.043, SE=0.097, p=0.658) nor the interaction between group and the condition (Coef=0.005, SE=0.033, p=0.884) reached significance. Similar to the accuracy data, age (Coef=-0.003, SE=0.007, p=0.645) and level of education (Coef=-0.002, SE=0.025, p=0.933) had no significant effect on RTs. **Figure 3** shows the average reaction time for participants under two conditions, with each point representing a participant.

## 4 Discussion

In this study, the impact of methamphetamine abuse on sequential working memory function was investigated by applying a sequential working memory task to 62 individuals with methamphetamine dependence and 59 healthy controls. Results showed that individuals dependent on methamphetamine exhibited lower performance compared to the controls in the Random condition but not in the Ordered condition. This finding provides evidence for the hypothesis that methamphetamine abuse impairs the functioning of sequential working memory.

The present finding not only complete previous studies on the impact of methamphetamine abuse on working memory but also provide explanatory insights into the impact of methamphetamine on cognitive processes involving sequential information. Two previous studies have tested methamphetamine-dependent participants and normal controls with a behavior assessment kit covering a list of activities such as comprehension and planning, financial ability, communication skills, transportation, household skills, and medication management (23, 24). These activities had a common characteristic: completing some minor tasks sequentially. The authors found that methamphetamine dependence was linked to poor performance in these tasks. Based on the current finding, we propose that dysfunctional sequential working memory could serve as (one of) the underlying causes of the impaired task performance.

Although the neural mechanism of the impaired sequential working memory was not the target of the current study, integrating the present finding with those on patients with Parkinson’s Disease (PD) nevertheless allow us to gain valuable insights into this issue. PD is a neurodegenerative disorder characterized by the progressive degeneration of dopaminergic neurons located in the substantia nigra, resulting in the loss of dopamine primarily in the basal ganglia (25). While the symptoms may vary, individuals who are dependent on methamphetamine are at a greater risk of developing PD (26), possibly because both PD and methamphetamine abuse result in disruptions with the dopamine circuits and their cortical or cortico-striatal targets. One recent study examining the sequential working memory of PD patients using a task identical to the one employed in the current study yielded findings comparable to the present one, with the PD group exhibiting inferior performance compared to the control group in the Random condition but not in the Ordered condition (13). Given the involvement of dopaminergic systems in the functioning of sequential working memory (13, 14), which are also primary targets of neurotoxicity induced by methamphetamine abuse, we would like to suggest that the functional deficit in sequential working memory identified by this study stems from the dopaminergic system dysfunction caused by methamphetamine abuse. However, due to the extensive brain regions damaged by methamphetamine abuse, these considerations are purely speculative and require further neuroimaging evidence.

In conclusion, by employing a task tailored to isolate the functioning of sequential working memory and by testing a relatively large pool of participants with methamphetamine abuse, the current study demonstrates (again) that methamphetamine abuse has profound impacts on diverse aspects of working memory, including the sequential processing of information that has not been examined in previous studies.

## Data Availability

The original contributions presented in the study are publicly available. This data can be found here: https://osf.io/gfa3e/.
